# The association between maternal perinatal mental health and perfectionism: A systematic review and meta‐analysis

**DOI:** 10.1111/bjc.12378

**Published:** 2022-06-28

**Authors:** Clare Evans, Jana Kreppner, Peter J. Lawrence

**Affiliations:** ^1^ Sussex Partnership NHS Foundation Trust West Sussex UK; ^2^ Centre for Innovation in Mental Health School of Psychology University of Southampton Southampton UK

**Keywords:** anxiety, depression, mental health, perfectionism, perinatal

## Abstract

**Background:**

Perfectionism is an important feature of adult psychopathology. In the absence of a prior review of the role of perfectionism in perinatal psychopathology, we aimed to ascertain whether perfectionism was associated with symptoms of maternal perinatal depression and anxiety.

**Method:**

We followed PRISMA guidance (PROSPERO: 42019143369), estimated weighted effect sizes and tested possible moderators: timing (pre or post‐ natal), scales used to measure constructs, infant gender, temperament and age; and rated study quality.

**Results:**

Fourteen studies met eligibility criteria. Perfectionism as a whole, and the perfectionistic concerns dimension, were moderately correlated with common maternal perinatal mental health difficulties *r* = .32 (95% Confidence Interval = 0.23 to 0.42). In sub‐group analyses, perfectionistic concerns were associated with depression (*r* = .35, 95% CI = 0.26–0.43). We found no evidence of significant moderation of associations.

**Limitations:**

Included studies had methodological and conceptual limitations. All studies examined depression and two examined anxieties; all examined perfectionistic concerns and four examined perfectionist strivings.

**Conclusions:**

Perfectionism, namely perfectionistic concerns, is potentially associated with common maternal perinatal mental health problems. While further research is warranted, identification of perfectionism in the perinatal period may help focus resources for intervention, reducing the prevalence of perinatal mental health difficulties.

**Practitioner points**

- Perfectionism, especially perfectionistic concerns, might be important in the context of common maternal perinatal mental health problems.
- Accounting for perfectionism in assessment and formulation of common maternal perinatal mental health difficulties could fruitfully inform clinical psychology practice.

## INTRODUCTION

Depression and anxiety disorders are the most common mental health disorders in the perinatal period (Howard et al., 2014). Estimates for the prevalence of maternal perinatal depression range between 9% and 13% in high income countries (Gavin et al., 2005), with mothers in low and middle income countries at almost twice the odds of the disorder compared to mothers in high income countries (Parsons et al., 2012; Woody et al., 2017). Prevalence of anxiety disorders ranges between 3% and 39% prenatally, and 3% and 20% postnatally (Goodman et al., 2016; Vesga‐Lopez et al., 2008). Anxiety disorders and depression commonly co‐occur (Breslau et al., 1995; Leach et al., 2017), with two thirds of women with postnatal depression experiencing a comorbid anxiety disorder (Falah‐Hassani et al., 2016; Wisner et al., 2013). Symptoms of these disorders are the same perinatally as at other times, though triggers can commonly centre on themes related to child rearing (Misri et al., 2015). Experiencing perinatal mental health difficulties is associated with; higher risk of preterm delivery (Grigoriadis et al., 2013; Grote et al., 2010), negative changes in infant brain development (Lebel et al., 2016), delays in infant attainment of developmental milestones (Letourneau et al., 2013), lower child IQ (Barker et al., 2011) and disruptions in executive functioning (Buss et al., 2011), as well as dysregulated sleep and feeding in the infant (Sharkey et al., 2016). Symptoms can also affect mothers' ability to effectively nurture their child (American Psychological Association, 2013). Infants of mothers with perinatal mental health difficulties are at greater risk of being subject to care orders (Howard et al., 2004) and, in the most extreme cases, death (Sanderson et al., 2002).

In fact, perinatal maternal anxiety disorders and/or depression are associated with wide‐ranging adverse systemic outcomes. For children, these include behavioural disturbances (Murray et al., 2018), increased risk of anxiety disorders in childhood (Lawrence et al., 2020) and, in adolescence, poorer academic performance (Murray et al., 2010) and increased likelihood of depressive disorders (Netsi et al., 2018). For society, the economic and social cost of perinatal mental health disorders in the UK alone is estimated to be £8.1 billion a year (Bauer et al., 2014). Accurately identifying the risk factors for perinatal mental health disorders will improve screening precision, and effectiveness of prevention and intervention; therefore, reducing both the economic and emotional long‐term costs.

### Perfectionism, anxiety and depression

Perfectionism plays a central role in anxiety disorders and depression (Limburg et al., 2017) and, therefore, constitutes a putative therapeutic target in their prevention or treatment (Shafran et al., 2018). The relationship between perfectionism and common mental health difficulties (depression and anxiety disorders) can potentially be explained through Beck's three stage cognitive model of vulnerability (Beck, 1967). The first stage, negative childhood experiences, are likely to stem from the interaction between temperament (in the case of perfectionism, high levels of emotionality) and parenting approaches characterized by criticalness, excessive expectations, potential lack of care, over control and overprotectiveness (Enns et al., 2002; Flett et al., 2002). These negative childhood experiences lead to the development of dysfunctional beliefs about approval of others and self‐worth being contingent upon meeting expectations (Blatt, 1995). Negative childhood experiences leading to development of dysfunctional beliefs, consequently leave individuals vulnerable to psychological difficulties (Beck, 1967).

Perfectionism can be understood as setting exceptionally high standards leading to overly critical self‐evaluation (Frost et al., 1990). Cognitive behavioural theories of perfectionism suggest difficulties stem from, and are maintained by, self‐imposed dysfunctional standards (beliefs), continued striving towards and the adverse consequences of not meeting said standards (Shafran et al., 2002).

Perfectionism is now generally agreed by researchers to be multidimensional although it was originally conceived as a unidimensional construct (Burns, 1980). Factor analyses of the most commonly used measures of perfectionism (i.e., the Frost Multidimensional Perfectionism Scale ‐FMPS; Frost et al., 1990; and the Hewitt Multidimensional Perfectionism Scale ‐HMPS; Hewitt et al., 1991) typically yield a two‐factor solution: perfectionistic concerns and perfectionistic strivings (Bieling et al., 2004). The two factors appear to draw on different motivations. Perfectionist concerns centre around fears about activity being scrutinized by others, whereas perfectionist strivings are much more self‐focused, with standards individually imposed (Smith et al., 2015).

Perfectionistic concerns reflect a preoccupation with mistakes, excessive concerns about others' expectations and excessive negative reactions to perceived failures (Smith et al., 2015). Perfectionistic strivings are reflected in the ceaselessly demanding perfection of oneself. Perfectionistic strivings and perfectionistic concerns were by many viewed as mapping on to adaptive and maladaptive forms of the trait, respectively (Frost et al., 1993). Perfectionistic concerns have been associated with mental health symptoms and disorders with considerable frequency (Hewitt et al., 1991). Despite a paucity of research identifying relationships between perfectionistic strivings and mental health conditions, positive correlations with both depression and eating disorders have led to a move away from the simplistic understandings of perfectionism factors. Several researchers, therefore, now oppose the view that there are maladaptive and adaptive forms of perfectionism in favour of a position purporting that varying loads of either perfectionism factor, may increase the likelihood of mental health difficulties (Stoeber & Otto, 2006).

In the first meta‐analysis of the association between perfectionism and general psychopathology, Limburg et al. (2017) reported a weighted mean effect size of *r* = .26 (in samples unrestricted by age or particular periods of life). However, drawing on data from both clinical and non‐clinical samples, perfectionistic concerns and perfectionistic strivings were differentially associated with anxiety and depression throughout adulthood (Limburg et al., 2017). When considering clinical disorder, perfectionistic concerns (while controlling for perfectionistic strivings), were positively associated with anxiety disorders (ß = .33, 95% CI = .29 to .37) and depression (ß = .40, 95% CI = .32–.48). In contrast, perfectionistic strivings (while controlling for perfectionistic concerns), were negatively associated with anxiety clinical disorders (ß = −.08, 95% CI = −.12 to −.03) and not meaningfully associated with clinical depression (ß = .01, 95% CI = −.08 to .09). Similarly, when anxiety and depression symptoms (rather than diagnosable disorder) were considered, perfectionistic concerns, were positively associated with anxiety symptoms (ß = .36, 95% CI = .34 to .37) and depressive symptoms (ß = .42, 95% CI = .41 to .43). However, perfectionistic strivings, were negatively associated with both anxiety symptoms (ß = −.02, 95%, CI = −.03 to −.01) and depressive symptoms (ß = −.08, 95%, CI = −.09 to −.07). Research looking at symptom severity, as opposed to solely clinical diagnoses, helps to examine tendencies towards difficulties and prevents misclassification to ensure that prevention and treatment approaches focus on the full spectrum of struggles.

### Perfectionism perinatally

Perfectionism is likely to have particular relevance for maternal mental health in the perinatal period, but its role is currently not clear. Commonly held expectations regarding parenthood (Biehle & Mickelson, 2012), unrealistic ideals and romanticized views of motherhood (Douglas & Michaels, 2004) could be congruent with, and trigger, existing perfectionist traits. Existing research has focused more on the postnatal than prenatal period, and depression over anxiety (Egan et al., 2017; Gelabert et al., 2012; Grazioli & Terry, 2000; O'Hara et al., 1982; Thompson & Bendell, 2014).

Effect sizes for the association between perfectionistic concerns and postnatal depression symptoms range between 0.13–0.56 (Dimitrovsky et al., 2002; Oddo‐Sommerfeld et al., 2016). For perfectionistic strivings, some research suggests non‐significant associations with postnatal depression symptoms (Dimitrovsky et al., 2002; Maia et al., 2012), while other studies have reported significant negative correlations (Mazzeo et al., 2006). Further, findings from studies examining perfectionistic concerns in the prenatal versus postnatal period are inconsistent. One longitudinal study reported moderate to strong associations between perfectionistic concerns and common mental health conditions prenatally (anxiety *r* = .44, *p* < .01 and depression *r* = .56, *p* < .01) but the associations are notably lower postnatally compared to prenatally: anxiety *r* = .33, *p* < .01), depression *r* = .37, *p* < .01 (Oddo‐Sommerfeld et al., 2016). Differences in associations between perfectionistic concerns and mental health conditions during the prenatal and postnatal period were not, however, apparent in other studies (Maia et al., 2012).

Findings regarding the role of perfectionistic strivings in common mental health difficulties prenatally versus postnatally are also unclear. Macedo et al. (2009) reported a positive association prenatally with anxiety (*r* = .16, *p* < .001) but not depression (*r* = .11, *p* > .05), while Maia et al. (2012) reported a significant association with depression prenatally (*r* = .135, *p* < .001) but not postnatally (*r* = .072, *p* > .05). In summary, there is a clear need to systematically and quantitatively synthesize the current literature examining the role of perfectionism perinatally in maternal mental health.

### The current study

Our primary aim is to quantify whether maternal perinatal mental health is associated with perfectionism and, more specifically, whether maternal anxiety and depression (as defined in the DSM‐5, American Psychological Association, 2013) and identified as the most common difficulties in the perinatal period (Howard et al., 2014), are each differentially associated with perfectionistic concerns and perfectionistic strivings perinatally.

Our secondary aim concerns possible moderating factors. Although the risk literature focuses on genetic, environmental (including stressful life events), social, obstetric and infant factors, and more recently psychological factors (O'Hara & Wisner, 2014), this research aimed to focus on offspring factors; acknowledging the intertwined relationship between mother and baby during this period. Indeed, theory and empirical studies indicate that maternal anxiety (Murray et al., 2009) and depression (Goodman & Gotlib, 1999) are associated with offspring/infant factors including; age (Campbell & Cohn, 1997; Kitamura et al., 1993), gender (Tronick & Reck, 2009) and temperament (Britton, 2011).

These factors, as well as the particular measures used to examine constructs of perfectionism, maternal perinatal mental health and study design (cross‐sectional vs longitudinal); may moderate the strength of associations (Limburg et al., 2017). Thus, as a secondary aim, we will examine the following; impact of the time of measurement (prenatal or postnatal); infant age, gender and temperament, the measures used to assess perfectionism and maternal mental health, and study design; on the associations between perfectionism sub‐domains with maternal perinatal anxiety and depression.

## METHODS

### Protocol

We adhered to the preferred reporting items for systematic reviews and meta‐analyses (PRISMA) guidance (Page et al., 2021) and pre‐registered our review on PROSPERO (protocol number: CRD42019143369). The protocol was registered after preliminary searches were conducted and the study registered on the Open Science Framework platform for transparency. All data and code for analyses reported in this paper are available on the OSF: https://osf.io/ysmjv/.

### Eligibility criteria

See Table 1.

**TABLE 1:** Eligibility criteria for papers included in the review

Inclusion criteria	Exclusion criteria
Patient population	
Participants human, female, at least 18 years	Participants with co‐existing severe mental health issues (psychosis, bipolar)
Participants in perinatal period	
	Studies recruiting infants with additional needs (such as prematurity, congenital heart or problems or complex physical health needs)
Interventions	
Studies using any intervention; baseline data must be available.	
Comparators	
Studies using any comparison groups but data of interest only perinatal women.	
Outcomes	
Studies reporting perfectionism AND depression and / or anxiety symptoms (regardless of diagnostic status)	Solely qualitative
Perfectionism (either general or parenting specific)	
Measures used to assess anxiety, depression and perfectionism, must be validated.	
Data to be in format whereby Pearson’s *r* can be computed.	
Study design	
Studies including all designs (except retrospective)	Conference papers, posters and reviews
Written in English, German, Spanish or translation	Translated article unavailable
Published in peer‐reviewed journal	Unpublished research or book Ch.
	Qualitative study

### Information sources and search terms

**FIGURE 1:**
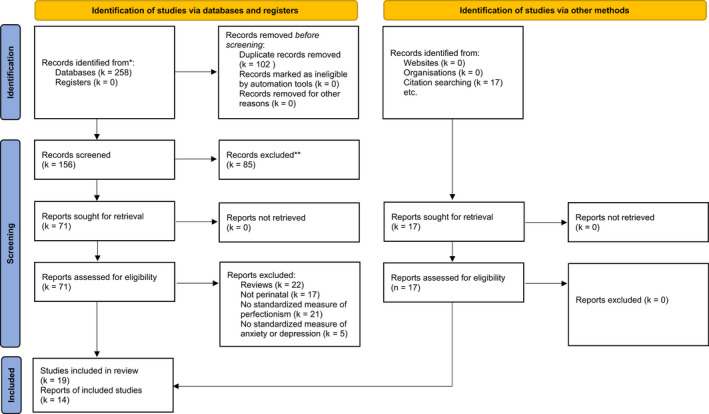
PRISMA flowchart of studies included in review

We searched six electronic databases for relevant published literature: Psycinfo (via the EBSCO interface), Cumulative Index of Nursing Allied Health Literature (CINAHL, through EBSCO), Medline (through EBSCO), EMBASE (via Ovid), Web of Science and PubMed originally on the 10th October, 2019 and repeating these on the 28th December, 2020, to capture more recent publications. We applied no time limitations or methodological search filters on any databases. We chose our search terms in consultation with a University Psychology Research Engagement Librarian and focused on the PICOS criteria (participant, intervention, comparison, outcome and study design; Harris et al., 2013). We used three main groups of terms to capture our main concepts; (1) perinatal period, (2) symptoms of common mental health issues and (3) perfectionism. Each search term was expanded to include as many possible cited variations of the construct. Medical Subject Headings (MeSH) terms were used in the Medline, Psycinfo, CINAHL and Embase databases, but not in PubMed or Web of Science due to lack of availability (The full search syntax used for each database is available in the Table S1). We hand searched the references of papers maintained for full text review (through a process of backward chaining), to identify other relevant papers to be assessed for inclusion in our review (see Figure 1).

### Outcome measures

We stipulated that all measures of depression, anxiety and perfectionism were validated. Measures of perfectionism had to map on to the perfectionist concerns and / or strivings dimensions (Table S2). All data had to be reported in a format allowing for computation of Pearson's *r* (see Table 1 ‐ full eligibility details).

### Study selection

Two authors (removed for blind review) independently screened titles and abstracts. A conservative approach was taken: where either reviewer did not exclude on the basis of abstract or title, the paper was taken forward for full text review. The same authors conducted the full text screening and, where discordant, discussed disagreements with the removed blind review third author, so that consensus could be reached. We excluded unpublished data and dissertations given the recognized difficulties in searching systematically (Egger et al., 2003).

### Study quality assessment

We used the QualSyst appraisal tool (Kmet et al., 2004). This tool provided a good fit with our review; research, which made no methodological exclusions beyond stipulating that studies collected quantitative data. The checklist contains 14 items (three of which focus on intervention trials, so were irrelevant to the present study), rated either 0 (no, not met), 1 (partially met) and 2 (yes, met), and a summary score is then produced. Given the absence of randomized controlled trials, we planned to exclude items 5, 6 and 7, which relate to random allocation and blinding. In the case of cross‐sectional designs, item 12 (‘were confounders controlled for?’) was also to be excluded. Items included questions on: clarity of question/objective, appropriateness of study design, appropriate method and description of variables, description of subject characteristics, definition and measurement of outcomes, appropriateness of sample size, analytic methods used, estimates of variances reported, controls for confounders, detail of results and conclusions appropriate. Quality assessments were carried out by two researchers (Clare Evans and Peter Lawrence), with discussion and consensus reached. Quality assessment summary scores can be found in Table 2.

**TABLE 2:** Study characteristics

References	Country	N	Age (*M*, *SD* or range)	Infant gender	Source	Design	Measurement points	Temperament	Quality score
Dimitrovsky et al. (2002)	Israel	100	*m* = 27.9 (no *SD*)	Not Reported	Community	Cross‐sectional	Antenatal	Not Reported	0.9
Hassert et al. (2018)^a^	Czech Rep	126	m = 30.30 (*SD* = 3.99)	Not Reported	Community	Cross‐sectional	Postnatal	Not Reported	0.8
Hassert et al. (2018 ^b^	Thailand	161	m= 25.83 (sd= 3.46)	Not Reported	Community	Cross‐sectional	Postnatal	Not Reported	0.8
Schoppe‐Sullivan et al. (2017)	United States	127	m = 27.80 (*SD* = 3.77)	Not Reported	Community	Prospective	Antenatal & Postnatal	Not Reported	0.77
Choi and Hyun, (2019)	Korean	150	Range: <30 *n* = 53, ≥30 *n* = 97	Not Reported	Community	Cross‐sectional	Postnatal	Not Reported	0.7
Grazioli and Terry (2000)	Australia	65 & 57 follow up	m = 28. 81 (*SD* = 3.36)	Not Reported	Community	Prospective	Antenatal & Postnatal	Bates 7 item (m= 3.44, sd =0.97)	0.77
O'Hara et al. (1982)	United States	170	m = 26.6 (no *SD*)	Not Reported	Community	Prospective	Antenatal & Postnatal	Not Reported	0.73
Church et al. (2005)	Australia	406	m = 29.2 (*SD* = 5.07)	Not Reported	Community	Cross‐sectional	Postnatal	Not Reported	0.85
Egan et al. (2017)	Australia	71	m = 32.3 (sd= 3.74)	Not Reported	Community	Prospective	Antenatal & Postnatal	Not Reported	0.9
Gelabert et al. (2011)	Spain	309	m =31.6 (sd=4.7)	Not Reported	Inpatient Obstetric	Prospective	Postnatal – 3 points	Not Reported	0.73
Gelabert et al. (2012)	Spain	122	m = 33.7 (*SD* = 4.10)	Not Reported	Inpatient Psychiatric	Prospective	Postnatal – perf when PND remitted.	Not Reported	0.95
Macedo et al. (2009)	Portugal	421	m = 29.8 (*SD* = 4.48)	Not Reported	Community	Cross‐sectional	Antenatal	Not Reported	0.86
Maia et al. (2012)	Portugal	386	m = 30.08 (*SD* = 4.21)	Not Reported	Community	Prospective	Antenatal & postnatal	Not Reported	0.9
Oddo‐Sommerfeld et al. (2016)	Germany	297, 266 follow up	m = 32.35 (*SD* = 4.46)	Not Reported	Community	Prospective	Antenatal & Postnatal	Not Reported	0.95
Thompson and Bendell (2014)	United States	77	m = 24.6 (*SD* = 4.72)	Not Reported	Community	Cross‐sectional	Postnatal	Not Reported	0.85

Hassert et al. (2018 ^a^ & Hassert et al. (2018 ^b^, same study but two different cohorts.

Abbreviation: *SD*, standard deviation.

### Data extraction and statistical analyses

Removed for blind review we extracted outcome data, as well as study characteristics (summarized in Table 2). We wrote to authors of eight papers; who did not report data in a format we could use to calculate effect sizes. Given our aim of understanding the relationship between perfectionism and common perinatal mental health symptoms, we chose zero‐order correlation coefficients (*r*) as our effect size (ES). For the purpose of this type of meta‐analysis, standardization of correlation coefficient *r* is recommended to obtain summary effects, confidence intervals and account for variance (Borenstein et al., 2009). Fisher's transformation of effect size *r* to *z* was performed for analyses, and then, to help with interpretability, results were transformed back to *r* (Borenstein et al., 2009). We used the Rstudio (Rstudio Team, 2015) to conduct analyses; specifically the ‘metafor’ package for meta‐analysis, the ‘weightr’ package for weighted sensitivity analyses (Viechtbauer, 2010) and the ‘robumeta’ package for meta‐analyses of dependent effect sizes (Fisher & Tipton, 2015). We estimated heterogeneity of effect sizes using *Q and τ* ^2^ and the *I* ^2^ statistic to calculate the impact of heterogeneity of effect sizes between studies and conducted meta‐regression (mixed effects meta‐analyses) to assess for moderation of effects. Ten included studies reported more than one outcome meaning effects would not be independent of each other. To address data dependencies, we used robust variance estimation to account for within‐study dependencies. Robust variance estimation does not require information on true correlation and thus, in line with recommendations (Tanner‐Smith et al., 2013), *τ* ^*2*^ was estimated with ρ = .80 in all analyses. The ‘robumeta’ package used in analyses for dependent effect sizes applies the Satterthwaite approximation (Satterthwaite, 1946) to adjust for small samples. When undertaking independent effects meta‐analyses, degrees of freedom (df) vary with the number of studies. When undertaking robust variance estimation (RVE) meta‐analyses of *dependent* effects, however, df also vary in relation to features of covariates. Hence, it is necessary to correct the degrees of freedom. When this corrected df value is below 4, the *p* value accompanying the hypothesis test is meaningfully likely to under‐estimate type I error, so it unreliable (Tanner‐Smith et al., 2016). In order to analyse the moderation effects of measures used for common mental health problems and perfectionism, we needed to create reference categories for both moderator variables because df < 4.

Following (Higgins & Green, 2011), we undertook moderator analyses when we had a minimum of 10 studies.

### Publication bias

We assessed for publication bias with Egger's test (Egger et al., 1997) and visual inspection of funnel plot to detect asymmetry (Borenstein et al., 2009).

### Moderators

Moderators included three categorical variables (timing: pre‐ or postnatal, psychometric measure used and study design) and three continuous (infant age, gender – reported as % of each, and temperament). Meta‐regression using robust variance estimation was used to examine moderators of the associations between maternal perfectionism and mental health (Hedges et al., 2010).

## RESULTS

In sum, 19 studies met our inclusion criteria (Figure 1 shows the PRISMA flowchart of included studies). We extracted data directly from 11 studies and requested data from the authors of the eight remaining papers. Two authors (of three papers) responded with additional data, leading to a total of 14 studies, comprising 40 effect sizes from 2988 participants, eligible for meta‐analysis (Table 3).

**TABLE 3:** Summary of results

References	Key outcomes	Type of perfectionism measured	Mental health measurement	Correlation (Effect Size *r*)
Dimitrovsky et al. (2002)	HMPS, DEQ (Anaclitic & Introjective)	Perfectionistic concerns & strivings	Depression	DEQ Anaclictic &: SOP MPS (*r* = −.15, ns), & SPP (*r* = .13, ns).
				DEQ Introject & SOP (*r* = −.13, ns), SPP (*r* = .40***)
Hassert et al. (2018 ^a^	DAS‐A‐17 (11‐items), EPDS	Perfectionistic Concerns	Depression	DAS‐A‐17 & EPDS (*r* = .636**)^1^
Hassert et al. (2018 ^b^	DAS‐A‐17 (11‐items), EPDS	Perfectionistic concerns	Depression	DAS‐A‐17 & EPDS (*r* = 0.392**)^1^
Schoppe‐Sullivan et al. (2017	SOPP subscale MPPQ, CES‐D	Perfectionistic concerns	Depression	SOPP &EPDS @ 3 months: (*r* = .23*) @9 months: (*r* = .02, ns)
Choi and Hyun (2019)	FMPS: COM, DAA & EPDS.	Perfectionistic concerns	Depression	EPDS &: COM (*r* = .49**), DAA (*r* = .40**)
Grazioli and Terry (2000)	DAS‐A (25 items) PE & AO, M‐DAS‐PE & AO & EPDS	Perfectionistic concerns	Depression	DAS‐PE &EPDS antenatal (*r* = .21, ns), postnatal = (*r* = .34*), DAS‐A0 & EPDS antenatal (*r* = .23*), postnatal (*r* = .37**), M‐DAS‐PE & EPDS antenatal (*r* = .17, ns), postnatal (*r* = .18, ns),M‐DAS‐AO & EPDS, antenatal (*r* = .02, ns), postnatal (*r* = .16, ns)
O'Hara et al. (1982)	DAS 40 item, BDI	Perfectionistic concerns	Depression	DAS & BDI (*r* = −.283****)^2^
Church et al. (2005)	DAS 24 item, EPDS	perfectionistic concerns	Depression	EPDS & DAS (*r* =.52**)
Egan et al. (2017)	EPDS, CPQ	Perfectionistic concerns	Depression	EDPS & CPQ antenatal (*r* = .36**), EPDS & CPQ postnatal (*r* = 27*).
Gelabert et al. (2011)	EPDS, FMPS scales: PS, COM, DAA	Perfectionistic concerns & strivings	Depression	EPDS week 8 &: PS (*r* = .271**), COM (*r* = .423**), DAA (*r* = .348**), EPDS week 32 &: PS (*r* = .325**), CM (*r* = .452**), DAA (*r* = .424**)^3^
Gelabert et al. (2012)	EPDS, FMPS	Perfectionistic concerns & strivings	Depression	EPDS week 8 &: PS (*r* = .131, ns), O (*r* = −.033), COM (*r* = .098, ns), DAA (*r* = .178*)^4^
Macedo et al. (2009)	BDI‐II & POMS Anxiety, HMPS SOP and SPP subscales (SPP divided SPP‐OHS, SPP‐CA)	Perfectionistic concerns & strivings	Depression & anxiety	SPP &: POMS anxiety (*r* = .24**), BDI‐II (*r* = .23**). SOP &: POMS anxiety (*r* = .16**), BDI‐II ns. SPP‐OHS &: POMS Anx (*r* = .20**), BDI‐II (*r* = .19**). SPP‐CA &: POMS anxiety (*r* = .18**), BDI‐II (*r* = .18**)
Maia et al. (2012)	BDI‐II & HMPS SOP, SPP subscales (SPP divided SPP‐OHS, SPP‐CA)	Perfectionistic concerns & strivings	Depression	Antenatal BDI‐II‐&: SOP (*r* = .135**), SPP‐OHS (*r* = .201**), SPP‐CA (*r* = .148**). Postnatal BDI &: SOP (*r* = .072 ns), SPP‐OHS (*r* = .212**), SPP‐CA (*r* = .212**)
Oddo‐Sommerfeld et al. (2016)	BDI‐V, EPDS, STADI, DP (Sum FMPS COM & DAA)	Perfectionistic concerns	Depression & anxiety	Antenatal DP &: BDI‐V (*r* = .56**), STADI Anx (*r* = .44**).Postnatal DP &: EPDS (*r*= .37**), Anx (*r* = .33**)
Thompson and Bendell (2014)	EPDS, HMPS SPP	Perfectionistic concerns	Depression	EPDS & HMPS SPP (*r* = .354**)

Abbreviations for Mental Health Measures: (1) DEQ (Depressive Experiences Questionnaire), (2) EPDS (Edinburgh Postnatal Depression Screener), (3) BDI (Beck Depression Inventory) various versions, (4) POMS (Profile of Mood States), (5) STADI (State‐Trait Anxiety Depression Inventory), (6) CES‐D (Center for Epidemiological Studies Depression Scale). Abbreviations for Perfectionism Measures: (1) HMPS (Hewitt Multidimensional Perfectionism Scale): SOP (Self‐Oriented Perfectionism), SPP (Socially Prescribed Perfectionism), SPP‐OHS (Socially Prescribed Perfectionism‐ Others High Standards), SPP‐CA (Socially Prescribed Perfectionism‐ Conditional Acceptance), (2) FMPS (Frost’s Multidimensional Perfectionism Scale): PS (Personal Standards), COM (Concern Over Mistakes), DAA (Doubt About Actions), (3) DAS (Dysfunctional Attitudes Scale), various versions including 11 items, 40 items and 25 items (including Performance Evaluation and Approval of Others elements), (4) M‐DAS (Maternal Dysfunctional Attitudes Scale) including PE (Performance Evaluation) and AO (approval of Others), (5) SOPP MPPQ (Societal Prescribed Parenting Perfectionism of the Multidimensional Parenting Perfectionism Questionnaire) (6) CPQ (Clinical Perfectionism Questionnaire), (7) DP (Dysfunctional Perfectionism scale‐includes elements of both FMPS COM & DAA).

^1,3,4^Correlational data received on request. ^2^Correlation x‐1, as DAS scale reverse scored (higher scores equalled higher functioning).

* *p* < .05, ** *p* < .01, *** *p* < .001, **** *p* < .0001.

**FIGURE 2:**
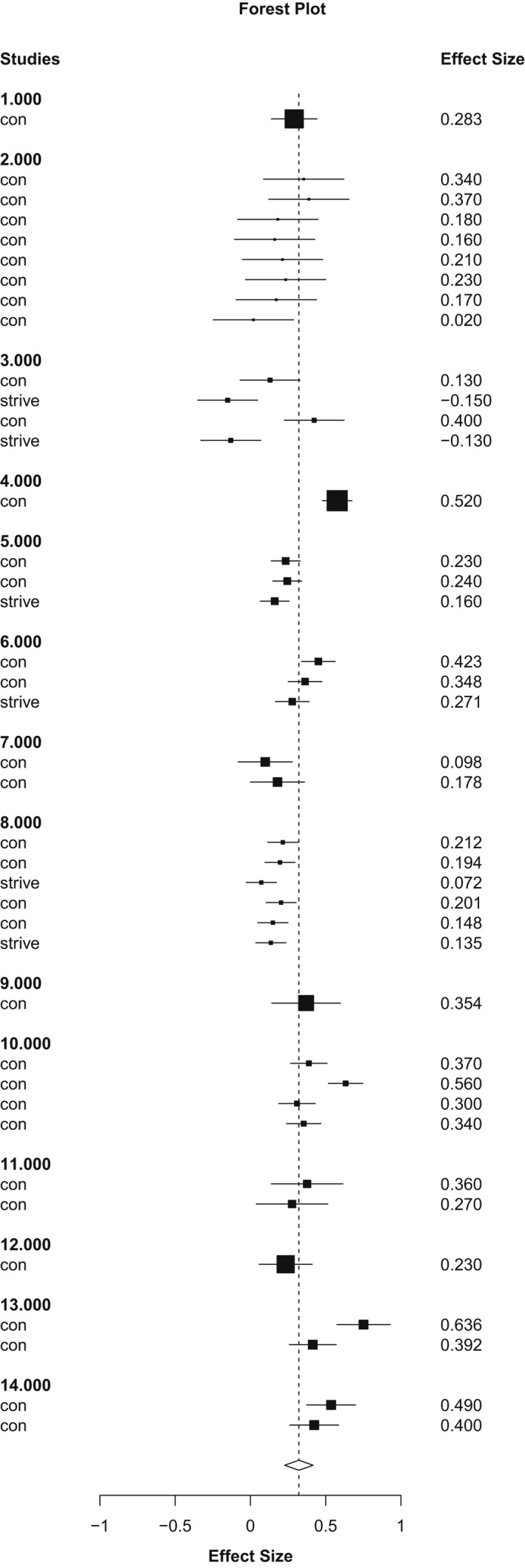
Forest plot of individual study effect Sizes & Pooled Effect Size of the associations between overall perfectionism and perinatal mental health using z scores (converted to r in report)

**FIGURE 3:**
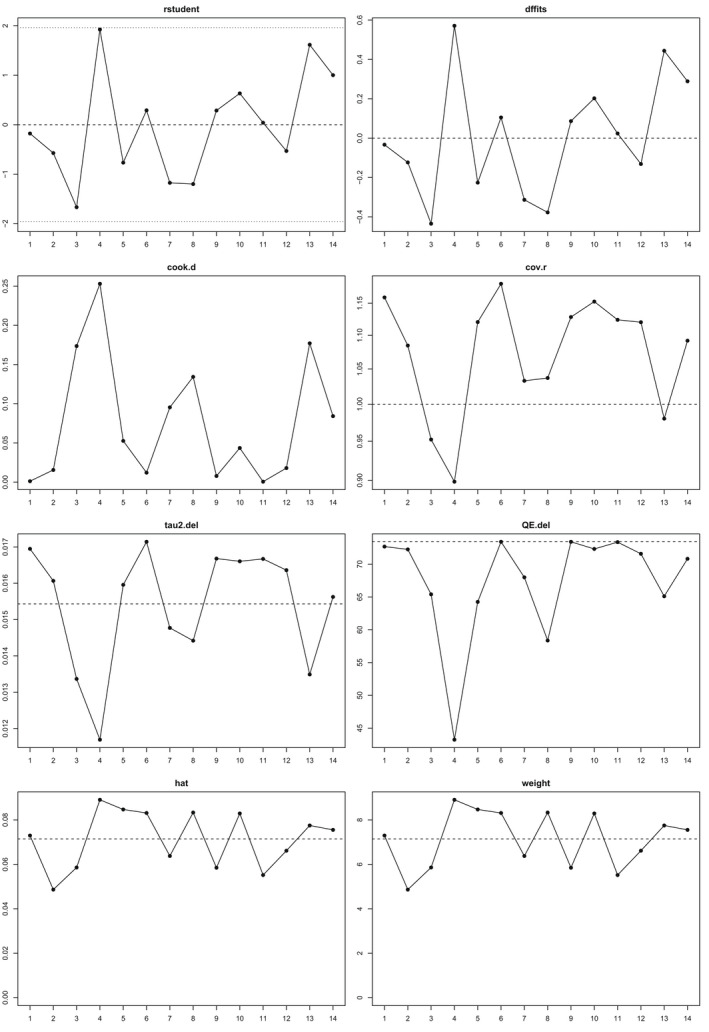
Leave‐one‐out diagnostic tests for influential studies

The overall weighted mean effect size (Figure 2) for the association between maternal perinatal mental health symptoms and perfectionism (that is, not distinguishing between depression or anxiety symptoms, nor perfectionism dimensions), was *r* = .32, *k* (number of studies) = 14, *es* (number of effect sizes) = 40, *p* < .01, 95% Confidence Interval (CI) = .23 to .42). We used random effects meta‐analysis to investigate heterogeneity. First, we calculated mean effect sizes for each study (without distinguishing between perfectionism dimensions). The *Q* test revealed the presence of heterogeneity (*Q* [13.10] = 87.01, *p* < .001), *τ* ^*2*^ = .03 (Standard error = .01), an *I* ^2^ of 84.9% indicating that heterogeneity had a significant impact on outcomes. We, therefore, consulted the influence plots (Figure 3) for further evidence and to examine whether individual studies were outliers. No individual study outliers were found, reducing concern over heterogeneity.

**FIGURE 4:**
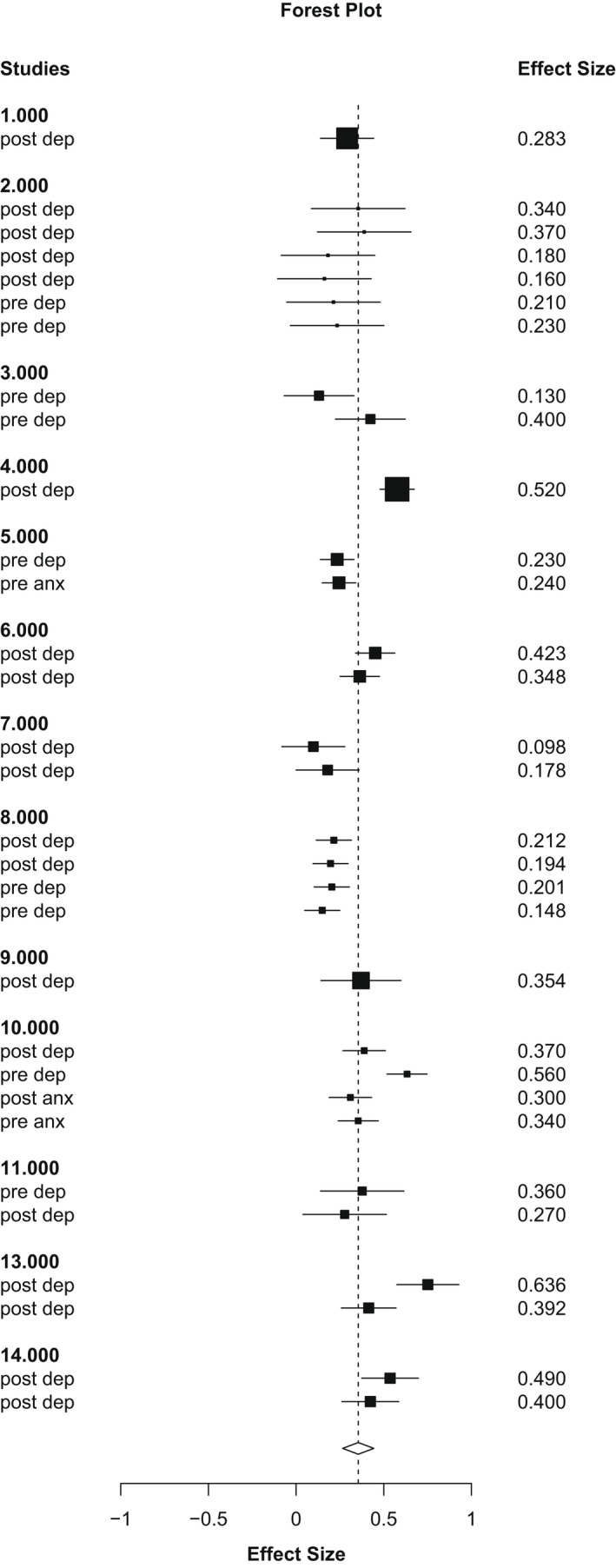
Forest plot of study effect sizes & pooled effect size for the association between perfectionistic concerns and maternal perinatal mental health symptoms as z scores (converted to r in report)

There was a significant association between perfectionistic concerns and perinatal mental health (*r* = .34, *k* = 14, *es* = 32, 95% CI = .26–.43; see Figure 4). There were insufficient studies to look at the relationships between perfectionism and anxiety alone. Furthermore, there was only one degree of freedom in analysis of perfectionistic concerns and anxiety outcomes, so we could not calculate a reliable pooled estimate.

We were unable to calculate a reliable pooled effect for perfectionistic strivings because degrees of freedom were 2.92. Hence, we conducted no analyses of moderation of associations between perfectionistic strivings and any outcomes.

### Moderation analyses

We were able to conduct moderation analyses for associations between perfectionistic concerns and depression outcomes for: study design (cross‐sectional vs longitudinal), timing of maternal depression measurement (ante‐ vs. postnatal), and measure used to assess depression (not anxiety due to insufficient study numbers).

We found no evidence that the association between maternal depression and perfectionistic concerns was moderated by study design (cross‐sectional vs longitudinal, *t*[5.4] = −.25, *p* = .81) nor by timing of measurement of maternal mental health (antenatal vs postnatal, *t*[6.55] = .09, *p* = .23). In our initial analysis of whether the measure used for maternal perinatal mental health problems significantly moderated the association between maternal mental health and perfectionistic concerns, degrees of freedom were below four. Hence, we created a theoretically coherent reference category comprising the most commonly used measure (the Edinburgh Postnatal Depression Scale, EPDS) and conducted a categorical moderation analysis of EPDS vs ‘Other’ (‘Other’ included any other measure of depression and no measures of anxiety). The measure of depression was not a significant moderator (*t*[11.56] = −1.90, *p* = .83). Too few studies reported infant age (*k* = 5), gender (*k* = 0) or temperament (*k* = 1), and no two studies used the exact same measure of perfectionism, so we could not conduct meta‐regression for these putative moderators.

### Study quality

The final studies selected for the review (*k* = 14), were quality assessed for bias using the QualSyst appraisal tool (Kmet et al., 2004). As previously stated, we excluded items 5, 6 & 7 and in the case of cross‐sectional designs, item 12 (‘were confounders controlled for?’) was also excluded. This meant total scores were either out of 20 or 22, quality scores between 0.00–1.00 were then generated from total scores. The quality rating of one study was based on the English translation (Choi & Hyun, 2019). Regarding rating study quality, interrater reliability was good (Cohen's k. .779, 95% CI = .632 to .925). Studies were generally highly rated in four domains: evident and appropriate designs; descriptions of participant characteristics; definitions of disorder diagnostic tools; and quality of diagnostic tools. The main area of concern was that 11 studies were scored as only ‘partially addressing’ their methods of participant selection. Two studies rated as .95, had strong methodological rigour. A summary of quality ratings can be found in Table 2.

### Sensitivity analysis

Sensitivity analysis of all studies was carried out. The smallest effect size (Dimitrovsky et al., 2002), as well as the largest (Church et al., 2005), were removed to try to understand their relative impact on weighted average effects. Leave‐one‐out sensitivity analyses yielded a smallest effect size of *r* = .2978 (*p* < .001, 95% CI = .22 to .36, *I* ^2^ of 71.56%) and a largest effect size of; *r* = .3369 (*p* < .001, 95% CI = .26 to .41, *I* ^2^ of 76.86%); indicating that no single study had a notable impact on the overall effect size.

Visual inspection of the funnel plot (Figure S2) suggested possible asymmetry, and Egger's test supported the presence of significant asymmetry (z = −1.97, *p* = .049).

## DISCUSSION

Our primary aim was to quantify whether maternal perinatal mental health is associated with perfectionism and, more specifically, whether perinatal anxiety and depression are each differentially associated with perfectionistic concerns and perfectionistic strivings. We included 14 studies with 40 effects from 2988 participants and found that maternal common perinatal mental health conditions of depression and anxiety (primarily depression, anxiety was reported in only two studies) are significantly associated with the overall perfectionism construct and the perfectionistic concerns sub‐domain. Maternal perinatal mental health difficulties were significantly associated with overall perfectionism, *r* = .31 (95% CI = .22 to .39), indicating a medium effect size (Cohen, 1992), comparable with the findings from Limburg et al. (2017) examining this association throughout adulthood (*r* = .26, 95% CI = .25 to .29). We examined perfectionistic concerns and strivings separately. The weighted average effect size for the association between trait perfectionistic concerns and perinatal mental health symptom severity was significant (*r* = .34, 95% CI = .26 to .43). We were unable to draw firm conclusions regarding the association between the perfectionistic strivings factor and maternal perinatal mental health (depression and/or anxiety) because too few studies reported data for us to analyse. Meaningful examination of moderators was only possible for the association between perfectionistic concerns and maternal mental health, specifically for the variables of timing, mental health measure (EPDS or Other) and study design (cross‐sectional versus longitudinal). None of these variables was found to significantly moderate the association between perfectionistic concerns and maternal mental health.

### Links to published research

Findings support the relevance of perfectionism during the perinatal period with associations between perfectionism and mental health difficulties indicated. The perinatal period is a time of transition (Miller & Sollie, 1980), during which new challenges are presented (Ventura & Boss, 1983) and distinct changes lead to understandable vulnerability (Bernstein et al., 2008; O'Hara & Wisner, 2014). At this time, perfectionistic traits may increase mothers concerns about their ability to be a good mother and to worry about their babies' ‘imperfections’ (Buist, 2006), potentially increasing vulnerability to mental ill health. Our meta‐analysis is consistent with previous studies that found significant positive associations between perfectionism and perinatal mental ill health (Church et al., 2005), including studies beyond the scope of our analysis, that found relationships both retrospectively (Mazzeo et al., 2006) and through single‐item screens (Milgrom et al., 2008). More specifically, our findings support the particular relevance of the perfectionistic concern dimension and its positive associations with perinatal mental health difficulties (Dimitrovsky et al., 2002; Maia et al., 2012; Oddo‐Sommerfeld et al., 2016).

The perinatal period is characterized by the need to rapidly acquire new skills (Ventura & Boss, 1983). Those high in the perfectionistic concerns dimension in particular, are likely to possess a heightened awareness of societal standards and are more likely to compare themselves to others in their ability to make shifts (Smith et al., 2015); as a result subjecting themselves to more negative personal evaluation, potentially culminating in perceived failure and resultant distress.

Timing of measurement (pre‐ vs. postnatal) did not significantly moderate the strength of the association between maternal perfectionism and mental health. Our findings are consistent with studies that support the existence of similar associations between perfectionism and perinatal mental health, both in prenatal and postnatal periods (Maia et al., 2012).

### Strengths of study

Strengths of our study included pre‐registration, the use of a comprehensive and systematic literature search of multiple databases, clear inclusion and exclusion criteria (with particular attention paid to the conceptualisation and measurement of the perfectionism constructs), blinded screening and quality rating by two reviewers, and the use of meta‐analytic techniques to quantify effect sizes. A further advantage of our meta‐analysis was that it used comprehensive methods of random effects modelling with robust variance estimation (Hedges et al., 2010); allowing us to use multiple effects from individual studies while controlling for data dependencies and variance due to dependent sample characteristics.

### Limitations

Our study design and pool of studies did not allow us to infer any meaningful differences between perfectionism prenatally versus postnatally. Six of the 14 studies used cross‐sectional designs, limiting the inferences that could be made about the role of perfectionism as a risk or maintaining factor in perinatal mental health.

Although eight of the 14 studies employed prospective longitudinal designs (a more robust design), these studies did not all span from ante to postnatal periods, hence both our exploration and the data itself were limited.

Only two of the 14 studies included measures of anxiety, therefore, our findings provide little further information about whether perfectionism is associated with multiple mental health difficulties during the perinatal period (as is the case in more general adulthood psychopathologies; Limburg et al., 2017). Although a clear limitation, inclusion of anxiety within our analysis adhered to our pre‐devised and registered protocol. Despite our failure to generate any evidence for perfectionism in relation to perinatal anxiety, both the prevalence of anxiety disorders in the perinatal period, as well as high comorbidity with depression (Falah‐Hassani et al., 2016; Wisner et al., 2013), warrant its continued investigation.

We limited the scope of our review to anxiety disorders as classified in the DSM‐5 (American Psychological Association, 2013). Hence, both Obsessive–Compulsive Disorder (OCD) and Post Traumatic Stress Disorder (PTSD) were outside our scope. The perinatal period, however, is associated with increased risk of onset of both OCD (Brander et al., 2016) and PTSD (Yildiz et al., 2017). Furthermore, perfectionism is a common feature in OCD (Frost et al., 2002). Thus, further research examining perfectionism in features of the anxiety disorders, OCD and PTSD, is needed to improve our understanding the role of the construct as possible trans‐diagnostic risk factor in the perinatal period. Studies included within the meta‐analysis illustrated good methodological quality (.7 to .9). However, quality points were lost on the following indices; sample size, lack of clearly specified objectives, description of subject selection and definition and robustness of outcome measures.

Unfortunately, subscales measuring perfectionistic strivings were missing from many studies (only *k* = 4 studies reported this), limiting our understanding of the meaning of this dimension perinatally. This is unfortunate exactly because in the broader literature, the case is made for the importance of the measurement of both dimensions: perfectionistic concerns *and* strivings (Bieling et al., 2004; Limburg et al., 2017; Stoeber & Otto, 2006).

We found that study design (cross‐sectional vs longitudinal) did not significantly moderate the association between perfectionistic concerns and perinatal mental health difficulties. This finding may, however, have been impacted by individual study differences in both the timing of perfectionism measurement and the period constituting the ‘longitudinal’ design. As stated, not all the longitudinal designs spanned the pre to postnatal periods; two were, in fact, postnatal only. Seven of the eight longitudinal studies attempted to record perfectionism scores at a separate time to the examination of mental health difficulties, in order to obtain a more ‘stable’ score of the construct. However, obtaining these measurements either during pregnancy or, in one study once postnatal depression had remitted (Gelabert et al., 2012), is unlikely to have provided a bias free score of ‘stable’ perfectionism; due to the impact that the significant shifts that occur in women both during pregnancy (Leifer, 1977) and as a result of an experience of maternal mental health difficulties (Bagby et al., 2008), are likely to have. In order to both gain a true measure of ‘stable’ perfectionism and understand how this period influences it future research examining perfectionism longitudinally during the entire perinatal period is warranted. This may allow us to both understand whether there are meaningful changes in the construct upon becoming a parent, as well as its potential impact on mental health.

We are also impeded in our ability to generalize our findings because the majority of studies employed non‐purposive approaches to recruitment, and only one study used a random sampling method. The plethora of different measures used to understand both perfectionism, depression and anxiety, as well as the potential impact on sensitivity and specificity as a result translating tools, was also likely to have impacted effect sizes across included studies too.

We were only able to include 14 of the 19 originally identified studies in our meta‐analyses. Although repeated efforts were made to contact the authors of these five studies, lack of response meant that our findings only reflect studies with outcomes made available in computable format. Sample sizes in the majority of studies were also small (with participant numbers ranging from 65–421), and participants were all self‐selecting; further limiting external validity (Schouten et al., 2009). Opting to exclude unpublished data due to the lack of clarity over peer review status and risk of retrieving duplicate effect sizes, is also likely to have limited our pool of studies. In their meta‐analysis, Limburg et al. (2017) queried the electronic Perfectionism Network Mailing List to identify studies that were accepted to a peer‐reviewed journal but not published at the time of the literature search. Future research should consistently adopt such an approach, in order to increase eligible studies.

Within our meta‐analysis, we chose to use continuous variables instead of clinical cut offs. This prevented us from making inferences about the impact of perfectionism on clinically diagnosed conditions, however, by not dichotomising depression or anxiety we hoped to avoid misclassification, as well as examine tendencies towards difficulties too (as even sub‐clinical manifestations of psychological difficulties are relevant to prevention and treatment approaches).

Our study focused on measures found to specifically load on to perfectionistic concerns and strivings factors (Limburg et al., 2017). Self‐criticism measures map closely on to perfectionism (Dunkley et al., 2006), but we did not include these due to lack of clarity regarding how they map specifically onto the two‐factor perfectionism model.. Further research into scales measuring overlapping concepts; such as self‐criticism is required.

The research came from nine different countries (see Figure S1), however eight of these studies (with the exception of Korea) were derived from Western samples, indicating a continued domination of the literature from Western countries; limiting generalisability of findings cross‐culturally (Adair et al., 2002). Although each country represented in the meta‐analysis is still likely to have its own distinct cultural idiosyncrasies around perinatal practices (Onoye et al., 2016), as well as variations in how distress is conceptualized (Oates et al., 2004), anecdotal evidence implies that perfectionism is more pervasive in individualistic Western societies that value individual achievement (Walton et al., 2020). With this in mind, further research is warranted in non‐Western samples to further our understanding of both perfectionism and its association with perinatal mental health in these societies.

## CONCLUSIONS

This meta‐analysis provides the first quantitative systematic analysis of the association between perfectionism and common perinatal mental health difficulties. We can make cautionary conclusions that there are meaningful, moderate positive associations between maternal perinatal mental health difficulties and perfectionism as a whole, and between depression and perfectionistic concerns in particular. By examining associations, we are not, however, able to determine the directionality (Asamoah, 2014) and thus cannot provide conclusive evidence that it is perfectionism influencing perinatal mental health issues as opposed to the other way round.

Indications that perfectionism, and in particular, the dimension of perfectionistic concerns, are positively associated with perinatal depression, do still have both clinical and research implications. Findings support a clinical focus on both early identification and preventive interventions. It could be recommended that early screening and identification of perfectionism in addition to depression and anxiety, may help focus resources for early intervention; reducing the prevalence of perinatal mental health difficulties, and, in turn, mitigating the risk for negative long‐term consequences on both infant development and maternal wellbeing (Bauer et al., 2014).

Our meta‐analysis also underscores the need for further exploration of the role of personality traits in perinatal mental health (Boyce, 1994); in particular, highlighting the possible relevance of perfectionism and supporting assertions that it is likely to play a role in psychopathologies across developmental and transitional periods (Shafran & Mansell, 2001).

The meta‐analysis revealed both methodological and conceptual limitations of current studies that need to be addressed. Further research into the association of both perfectionistic concerns and strivings factors with both depression and anxiety, and spanning the pre‐ and postnatal periods, is warranted. Such research will help improve our understanding of the association of both components of perfectionism with perinatal mental health, whether there is stability or change in these constructs, and their association with one another over the course of pregnancy and into motherhood. Investigation of possible moderators of this relationship, for example infant temperament, gender and age is currently lacking but warranted. These moderators may provide additional information regarding the context and specific stressors in which perfectionism is triggered and individuals subsequently become vulnerable to mental health difficulties (Beck, 1976).

## AUTHOR CONTRIBUTIONS

**Clare Evans:** Conceptualization; formal analysis; investigation; project administration; writing – original draft; writing – review and editing. **Jana Kreppner:** Conceptualization; investigation; methodology; supervision; writing – original draft; writing – review and editing. **Pete Lawrence:** Conceptualization; data curation; formal analysis; investigation; methodology; software; supervision; writing – original draft; writing – review and editing.

## CONFLICTS OF INTEREST

All authors declare no conflict of interest.

## Supporting information


Appendix S1

## Data Availability

The data that support the findings of this study are available via the Open Science Framework: https://osf.io/ysmjv/
