# Supplementary material for: The association between maternal perinatal mental health and perfectionism: A systematic review and meta‐analysis
Source: Br J Clin Psychol. 2022 Jun 28;61(4):1052–74. doi: 10.1111/bjc.12378 (PMC9796248; doi:10.1111/bjc.12378)
Supplement: Supplementary file 1 — Appendix S1 [file BJC-61-1052-s001.docx]

Supplementary Data Online Supplement

Table S1. *Full Search Syntax*

**Database Syntax**

Embase postpartum OR postnatal OR perinatal OR antenatal OR prenatal OR prebirth OR "post partum" OR "post natal" OR puerperium OR "peri natal" OR "maternal" OR "post pregnancy" OR "ante natal" OR "pre natal" OR "pre birth" OR "pregnant wom#n" OR pregnan* OR "expect* mother*" OR (exp “perinatal period/”) OR (exp pregnancy/) OR (exp puerperium/)

AND

depress* OR "mood disorder*" OR "affect* ADJ disorder*" OR anxiety OR "anxiety disorder*" OR anxi* OR (exp anxiety/) OR (exp “anxiety disorder/”) OR (exp depression/) OR (exp “major depression/”)

AND

perfection* OR "high standard*" OR "high expectat*" OR "concern ADJ mistake*" OR "parental perfection*" OR ((parental ADJ2 high) ADJ2 (standard*OR expectat*)) OR "personal standard*" OR perfor* ADJ (qualit* OR doubt* OR fear*) OR (exp perfectionism/)

PubMed postpartum OR postnatal OR perinatal OR antenatal OR prenatal OR prebirth OR “post partum” OR “post natal” OR puerperium OR “peri natal” OR maternal OR “post pregnancy” OR “ante natal” OR “pre natal” OR “pre birth” OR “pregnant woman” OR “pregnant women” OR pregnan* OR “expect* mother*”

AND

depress* OR “mood disorder*” OR “affect* disorder*” OR anxiety OR “anxiety disorder*” OR anx*

AND

perfection* OR “high standard*” OR “high expectat*” OR “concern mistake*” OR “parental perfection*” OR “parental high standard*” OR “parental high expectat*” OR “personal standard*” OR “doubt quality* performan*” OR “fear* quality* performan*”

Wof S postpartum OR postnatal OR perinatal OR antenatal OR prenatal OR prebirth OR “post partum”OR “post natal” OR puerperium OR “peri natal” OR maternal OR “post pregnancy” OR “ante natal” OR “pre natal” OR “pre birth” OR “pregnant wom$n” OR pregnan* OR “expect* mother*”

AND

depress* OR “mood disorder*” OR affect* NEAR/1 disorder* OR anxiety OR “anxiety disorder*” OR anx*

AND

perfection* OR “high standard*” OR “high expectat*” OR “concern NEAR/1 mistake*” OR “parental perfection*” OR “parental high standard*” OR “parental high expectat*” OR “personal standard*” OR "perfor* NEAR qualit*" OR "perfor* NEAR doubt*" OR "perfor* NEAR fear*”

Note. *DE or ME or MH or exp / captures MeSH headings or explosion of terms, * indicates truncation to include all possible variations following the symbol, # denotes a wildcard (any letter can replace it), N or ADJ or NEAR indicates near to (/ followed by a number denotes how near), “” indicating two words to be searched together.*

Table S2. *Measures mapping on to two Major Dimensions of Perfectionism.*

**Measure Perfectionistic concerns Perfectionistic strivings**

Multidimensional Concern over Mistakes (COM) Personal Standard (PS)

Perfectionism Scale Doubts About Actions (DAA)

(FMPS; Frost et al., 1990).

Including variations of.

Multidimensional Socially prescribed perfection Self-oriented perfection

Perfectionism Scale (SPP); tendency to expect others to (SOP); set high standards for

(HMPS; have high standards of them. self, with motivation to

Hewitt et al., 1991). Including sub-scales for SPP of: reach.

Including variations of. Conditional Acceptance (SPP-CA);

being loved and accepted is contingent

on achievement, Others High Standards

(SPP-OHS); others hold high standards or

Expectations for the self (Campbell &

Paula, 2002)

Dysfunctional Attitudes Scale Self- critical perfectionism

(DAS; Weissman & Beck,

1978). Including variations

more recent shortened

versions.

Multidimensional Parenting Societal prescribed parenting Self-oriented parenting

Perfectionism Questionnaire perfectionism (SPPP); beliefs that perfectionism (SOPP); (Snell et al., 2005) society expects them to be personal belief that a perfect parent. should be a perfect parent.

Maternal dysfunctional Performance Evaluation &

Attitudes Scale (M-DAS; Approval by Others

Grazioli & Terry, 2000)

Almost Perfect Scale-Revised Discrepancy High standards

(APS-R; Ashby et al., 2001)

Clinical Perfectionism Perfectionism

Questionnaire (CPQ; Fairburn,

Cooper, & Shafran, 2003))


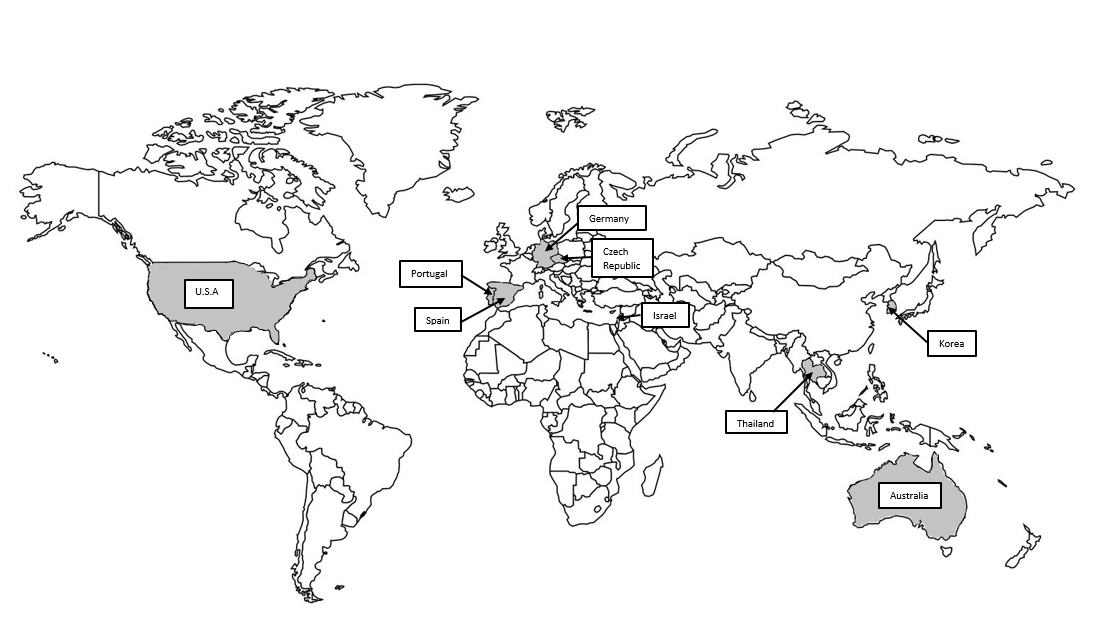


Supplementary Figure A. Map of the world with shaded areas indicating countries generating research included within meta-analysis.

Supplementary Figure B. Funnel plot indicating significant publication bias..
